# Physician perspectives on Choosing Wisely Canada as an approach to reduce unnecessary medical care: a qualitative study

**DOI:** 10.1186/s12961-018-0370-5

**Published:** 2018-09-26

**Authors:** Mark Embrett, Glen E Randall

**Affiliations:** 1 Centre for Health Economics and Policy Analysis, McMaster University, CRL, Building, 282, 1280 Main Street West, Hamilton, ON L8S4K1 Canada; 2 Health Policy and Management, DeGroote School of Business, McMaster University, Hamilton, ON Canada

**Keywords:** Health policy, unnecessary medical care, Choosing Wisely, shared decision-making

## Abstract

**Background:**

Reducing monies spent on unnecessary medical care is one possible target to improve value in healthcare systems. Regional variation in the provision of medical care suggests physician behaviour and patient demands influence the provision of unnecessary medical care. Recently, Choosing Wisely campaigns began using ‘top 5 do-not-do’ lists to target unnecessary medical care by encouraging greater physician and patient dialogue at the point of care. The present study aims to examine the rationale for Choosing Wisely Canada’s (CWC) design and to analyse physician perceptions regarding the features of CWC aimed to reduce unnecessary medical care.

**Methods:**

The study involved semi-structured interviews with 19 key informant physicians with CWC experience and the application of procedures of grounded theory to analyse interview transcripts and develop explanations addressing the objectives.

**Results:**

Participants reported that the CWC was the medical community’s response to three pressures, namely (1) demand for unnecessary medical care from patients during the clinical encounter; (2) public perception that physicians do not always prioritise patients’ needs; and (3) ‘blunt’ government tools aimed to reduce costs rather than improving patient care. Respondents stated that involving the patient in decision-making would help alleviate these pressures by promoting the clinical encounter as the paramount decision-point in achieving necessary care. However, CWC does not address several of the key reasons, from a physician perspective, for providing unnecessary medical care, including time pressures in the clinical encounter, uncertainty about the optimal care pathway and fear of litigation.

**Conclusion:**

This study contributes to our understanding of the perceptions of physicians regarding the CWC campaign. Specifically, physicians believe that CWC does little to address the clinical reasons for unnecessary medical care. Ultimately, because CWC has limited impact on physician behaviour or patient expectations, it is unlikely to have a major influence on unnecessary medical care.

## Background

In 2009, clinical experts from the Institute of Medicine in the United States estimated that 30% of healthcare costs were spent on unnecessary medical care [1]. An analysis of spending growth among United States regions revealed growing regional variations in healthcare costs between historically similar regions (in cost per capita spending). Similar concerns have been expressed in Canada [2]. Individual physician behaviour in the clinical encounter with patients is considered to play a substantial role in these variations [1]. Research indicates that physicians in high-cost regions are more likely to provide unnecessary medical care than those in low-cost regions [3]. Regional variation has been attributed to various causes, including, but not limited to, physician training in the same high-cost region in which they practice [4], remuneration methods, and access to technology and other resources [1].

In 2010, various medical specialty groups in the United States acted on this wastefulness. A proposal was made in the *New England Journal of Medicine* for each specialty area to develop evidence-based ‘top 5 do-not-do’ lists [5]. The ‘top 5’ lists were expected to be “*a prescription for how, within that specialty, the most money could be saved most quickly without depriving the patient of meaningful medical benefits*” ([5], p. 2). Shortly after the call for action, the National Physicians’ Alliance initiated the Promoting Good Stewardship in Medicine project to identify a list of five things that physicians could do in three specialties (family medicine, internal medicine, and paediatrics) in order to promote patient care and a more effective use of healthcare resources [6].

In April 2012, the American Board of Internal Medicine Foundation, in collaboration with *Consumer Reports*, built on the work of Brody and the National Physicians’ Alliance and formally launched the Choosing Wisely (CW) campaign in the United States, with ‘top 5 do-not-do’ lists from nine specialty societies, followed by 17 more societies producing lists 1 year later [7]. *Consumer Reports*’ role involved the distribution of campaign materials, including over 250 posters, brochures and wallet cards [8]. The CW campaign was designed to improve patient care by encouraging a conversation between the physician and patient at the point of care about not providing an unnecessary test or procedure included in the ‘top 5 do-not-do’ list such as distributing antibiotics for infections presumed to be a viral respiratory illness [9]. Unnecessary medical care may be defined as “*a diagnostic or treatment service that provides no demonstrable benefit to a patient*” ([10], p. 270).

Since the CW launch, there has been some scepticism expressed regarding the ability of the campaigns to reduce unnecessary medical care, including the impact on physicians’ knowledge of low-value care (which the campaign is intended to address), patients’ ability to ‘choose wisely’ in the context of medical care, and the inherent conflict of interest physicians have in choosing a low-value service that offers a high margin of remuneration [11]. Despite this scepticism about CW campaigns, many countries have begun adopting its design and have developed ‘do-not-do’ lists to promote within their own health system. Adopted by the University of Toronto and the Canadian Medical Association, the first to model the United States CW campaign, a Canadian version was initiated in April 2014 [12]. To begin, eight specialty societies developed and released their ‘do-not-do’ lists. At the time of writing, 25 societies have produced and published ‘do-not-do’ lists. The Choosing Wisely Canada (CWC) campaign has the same design and objectives as the CW campaign, which are to “*help physicians and patients engage in conversation about unnecessary tests, treatments, and procedures, and to support physician efforts to help patients make smart and effective choices*” ([13], p. 5).

CWC is promoted as a physician-led initiative that targets physician behaviour because the decision to determine the appropriateness of healthcare resources is, in most instances, ultimately the physician’s decision [13]. This study investigated physicians’ perceptions of the rationale for CWC design and its features aimed at reducing unnecessary medical care. The study aimed to obtain physicians’ perspectives regarding CWC to explain the rationale for CWC campaign’s design as well as to analyse physician perceptions of the features of CWC aimed at reducing unnecessary medical care.

## Methods

### Study design

To achieve the objectives of this study, we adopted a thematic qualitative analysis approach suited for smaller scale research studies aimed to develop grounded explanations that address the explanatory objectives of the researcher [14, 15]. As described by Corbin and Straus [16], these procedures, referred to generically as qualitative analysis of data, include a process of examining and interpreting data in order to elicit meaning, gain understanding and develop empirical knowledge, as opposed to theory.

### Recruitment

Based on the objectives of the study, it was deemed appropriate to recruit individuals who were key members of the groups who participated in CWC in order to “*maximize opportunities to develop concepts in terms of their properties and dimensions, uncover variations, and identify relationships between concepts*” ([16], p. 143). Therefore, we contacted members of all CWC medical groups, classified by CWC as medical specialty societies, medical organisations, provincial medical associations and CWC sponsoring groups, to request their participation in the study. Cross-referencing the CWC list with lists provided by the websites of the Canadian Medical Association and Canadian College of Physicians, we also identified associations that did not participate in the CWC with the intent to provide a balance of perspectives. All participants were contacted through email, with study information provided. Telephone follow-ups were conducted with some participants. Consent was obtained verbally before each interview. Ethics approval for the study was obtained through the Hamilton Integrated Research Ethics Board.

### Data collection

The primary investigator (ME) collected data through semi-structured interviews by phone between May and September 2015.

### Data analysis

Interviews were audio-recorded, transcribed, and then coded using the qualitative data analysis software NVivo. ME analysed all interview data. The components of this qualitative research design include methods to compare, code and report data to generate empirically ‘grounded explanations’ that address the objectives. Interview data and analytic memos were analysed together through three stages of coding [16, 17].

The first stage of the analytic process was open coding. During this stage, we analysed relevant data coded based on ME’s interpretation of the responses [18]. Transcripts were read and reread as data related to the rationale for, and features of CWC, were extracted and coded. During this stage, the relationships among codes began to emerge. In the second stage – axial coding – ME wrote descriptions of the data using the initial coding from interview data and analytic memos. These descriptions serve as the basis for the results of the study. As more transcripts were read, more codes emerged, and ME continued to make connections between them, using NVivo software as a tool. In the third stage – selective coding – we developed final explanations by comparing and reconstructing categories (made of various but similar codes) that were developed in the earlier stages. For example, categories for the rationale were compared and, if similar, they were synthesised into a clearer, ‘thicker’ explanation [19]. Saturation occurred when explanations that address the objectives were developed and participant responses began to be repetitive to those of previous participants and no new insights were revealed over the course of three consecutive interviews.

## Results

ME interviewed 19 key informants for the study and conducted 11 interviews with CWC representatives from different specialty societies. Participants that were contacted as members of medical associations and sponsor organisations had varied clinical specialties; however, since we did not focus on differences between specialties, we did not record their specific memberships. Societies and individuals that were contacted (*n* = 68), but did not participate (*n* = 49), either did not reply to recruitment emails or declined because they had no members that were sufficiently familiar with CWC. Other than those who worked for a CWC sponsoring agency, all participants were their association’s representatives for CWC, and many were society leaders. No differences in perspectives were identified among the participants’ responses, based on geographical location or specialty, when questioned about the reason for the CWC design and its features to reduce unnecessary medical care. Although the study participants included medical specialists from various disciplines, all participants from these groups are broadly referred to as physicians in this paper. Most interviews were approximately an hour in length, with one being 30 min.

The findings are organised by objective. Objective one is addressed in a single section, namely perspectives on the rationale behind the CWC design. Next, objective two (to identify and describe the CWC characteristics regarding reducing unnecessary medical care) is addressed in the two subsequent sections, namely the perspectives on positive features of CWC and perspectives on what drives unnecessary medical care in the clinical setting and why CWC does not fully address them.

## The rationale for the CWC design

Participating physicians reported that the medical community felt pressure to develop an initiative to reduce unnecessary medical care because of three factors, namely patient demand, public perception and government control. This section describes each of these pressures and explains how the design was perceived to address them.

### Patients are more informed and more demanding

All physician participants reported that patients often enter the clinical encounter with information and expectations about care. One physician spoke of their experiences with such patient demand:

“*There is a demand from patients for testing or medication or imaging that they’ve read about or they feel that they should get in order to be satisfied that they’ve been adequately cared for*.” (P001)

Another physician responded that patient demands are unsustainable:

“*Patients absolutely drive test ordering… Our society cannot go on with people walking into a clinic and demanding a service…*” (P002)

Easily accessible search engines and online users’ experiences were reported as the source of many of the patients’ information:

“*You know the rules… Google. Patients coming into a doctor’s office* [are] *armed with printouts from the Internet. Blogs are the chief example of the way that these things are shifting*.” (P003)

This respondent provides an example of patients bringing information into the doctor’s office to defend their perceived need for more care. A concern with this trend is that patients may not realise the potential for harm from overtesting, such as false positives, and from overtreatment, such as complications. This reflects a patient philosophy that many physician participants felt contributes to the rise of unnecessary medical care:

“*This* [information seeking] *feeds into the problem of screening: unnecessary screening,* [the] *harms that screening does, and the general perception that… patients… are demanding screening care, not realising that harms can be done*.” (P005)

Physician participants reported that the CWC campaign targets the clinical encounter as the optimal time to decide necessary care because patient inclusion in decision-making is expected to help reduce patient demand for an unnecessary service. One physician expected that using CWC materials would make it easier to demonstrate to patients that a service was unnecessary with CWC material:

“*It is easier to argue with the patient, saying... my Choosing Wisely says I should not.*” (P010).

The comment is speculative because even though physicians could envision how the patient encounter would unfold, only one participant had used CWC content in the clinical encounter. None of the participants had heard stories about others doing so, and many participants were unsure how many of their organisation’s members knew about the campaign. Furthermore, no physician participant reported adjusting aspects of the clinical encounter to better respond to patients’ demands for unnecessary medical care.

### The public: perceptions of a patriarchal physician

When asked why CWC was designed the way it was, participants reported that an initiative that improved public perception of the medical community was needed. Generally, physician participants reported that they perceived that many members of the public view the medical community as self-serving and motivated by factors other than patient care such as status and remuneration. This perception has affected physicians’ relationships with patients, as reflected by earlier findings that patients are seeking and bringing information into the clinical encounter to back up their claims of a needed service. Two physicians describe their perspectives on the public perception of physicians:

“*There has been a history of patronising or patriarchal physician behaviour that hasn’t really helped doctors’ cause.*” (P010)

“*I think, unfortunately, doctors to some extent come to be seen as self-serving, which makes their pronouncements less effective*.” (P001)

The CWC campaign attempted to address the public perception of the self-serving doctor. Incorporating patients into shared decision-making intended to promote a goodwill image of the physician:

“*This* [engaging patients in shared decision-making] *is an idea whose time has come; it’s very important. We’re* [patients and physicians] *seeing why unnecessary tests, treatments, and procedures are bad for quality care, so I think that there’s a lot of goodwill from physicians generally across Canada*.” (P003)

Removing costs from the CWC promotional strategy was intentional to help address some of the negative attitudes towards self-serving physicians by reducing remunerated care. Focusing on prioritising patient safety and highlighting the risks associated with over-providing was expected to keep the patient–physician discussion focused on how less care is sometimes better care, rather than being a cost control measure. Many physician participants did not feel patients wanted their physician to consider the cost of treatments when determining what medical care was appropriate.

### Governments: using blunt policy tools to remove medical services

Physician participants felt that the government has been restricting the availability of healthcare with ‘blunt’ policy tools that do not take patients’ health needs adequately into consideration. Instead, policies were primarily aimed at reducing costs. A physician noted:

“*There’s been certainly a lot of pressure on government to simply deny care… I think the government’s role has somewhat shifted, particularly in Ontario, around being more involved and has kind of moved towards steering, not rowing*.” (P003)

The preceding quote suggests that physicians perceive that the government is attempting to take a larger role in reducing unnecessary care. Another physician believed that the campaign was designed to take back control over what care is provided:

“*I think it’s part of this broader culture shift in medicine. Government initiatives can’t control what’s happening at the bedside and what’s happening clinically*.” (P014)

Part of the pressure identified by participants came from concerns that government decision-makers do not understand the clinical encounter enough to design effective policies to reduce unnecessary medical care. Some physicians commented on their mistrust for government policies because government does not understand the clinical needs of their patient:

“*Unless you are here doing the work, I don’t trust what you have to say*.” (P008)

Participant responses suggest that the CWC campaign was also a response to governmental efforts to remove services from the public purse, and positioned physicians as the proper authority on appropriate care. Physician participants stated that the medical community was in the right position to begin such a cultural shift away from government led initiatives and towards more self-regulation because they can have a conversation with patients about necessary medical care.

According to many physician participants, the accumulation of pressure from patients, the public and government resulted in the need for the CWC to embody this cultural change away from government-led initiatives. Leaders of the medical community adopted a strategy that would address these three pressures in one fell swoop by targeting the clinical encounter with improved patient dialogue.

## Positive aspects of CWC

When asked about whether the features of CWC are expected to be useful to reduce unnecessary medical care, participants identified the increased role of the patient, leadership by physicians and the simplicity of the process as potential benefits.

### Patient role is increased

As discussed, a major component of CWC is the dialogue it encourages between the physician and the patient about the patient’s care. Physician participants reported that engaging the patient in a conversation during the clinical encounter is critical to CWC achieving its set goals because an informed patient should make better choices about their medical care needs. Two physicians noted:

“*I think the patient is their best advocate…, but sometimes it’s misguided… There has to be a dialogue and that, I think, is very important.*” (P001)

“*I think patients have a really important role; they should question physicians more frequently about the tests that are being done*.” (P007)

Physician participants felt this type of bottom-up approach was a better way to reduce unnecessary medical care, rather than government-imposed policies:

“*Moving the conversation out of that sort of administrator* [or] *policy* [conversation]*… to something that I can deal with in* [a] *conversation I can have with a patient who is engaged in the conversation, rather than just doing a policy innovation*.” (P014)

All participants stated that patients ought to have an important role in determining their own medical care. This is envisioned through shared decision-making in the clinical encounter, which is a key characteristic of CWC.

### Physician-led initiative

For physician participants, it was important that peers in the medical community lead the CWC, not government, because the medical community generally perceives that government is more concerned with cost than the provision of necessary medical care:

“*I think there is an inherent distrust from the physician community to government and its ability to do things*.” (P005)

“[Physicians] *believe most of the decision-makers in* [government] *agencies are so far removed from patient care, that they are untrustworthy.*” (P007)

Participants were clear that there would have been very little, if any, buy-in by physicians if the government had an active role in the development or implementation of CWC. One participant described their medical society’s refusal to participate in CWC until they determined that it was not a government initiative. Government participants agreed that physicians did not often agree with government policies that regulated care:

“*Physicians do respond when there are kind of top-down changes to practice or kind of pushed on them by government, and that hasn’t always been positive*.” (P015)

### Easier accessing to evidence for shared decision-making

Participants reported that the format of CWC was very accessible because it provided simple messages that a physician could communicate to patients during the clinical encounter. As two physicians noted:

“*Choosing Wisely’s materials are very easy on the eye. It’s focused. This is what you need to know; this is how you are going to help explain it to your patient. It’s easy; it’s kind of sexy.*” (P007)

“*Choosing Wisely is not about* [revealing] *new evidence; it’s about somehow packaging it better*.” (P008)

When asked how CWC material compared to clinical guidelines, physician participants reported that CWC campaign material complemented guidelines with simpler, straightforward advice concerning what ‘not to do’ rather than ‘what to do’ situations. For example:

“*Nothing is stronger than saying ‘don’t do this’…. nothing is stronger than one professional society saying to another ‘stop the madness’, right? As opposed to listing situations where something is appropriate* [clinical guidelines]*, highlighting where it’s inappropriate is probably a little bit more attention grabbing than it being lost somewhere in the clinical practice guidelines that’s 704 pages*.” (P002)

This quote highlights that participants’ view that CWC addresses some shortcomings and complications of clinical guidelines. Many physician participants stated that they did not keep up with clinical guidelines because of their length and complexity, and that CWC materials are much easier to use.

## Shortcomings of CWC: not addressing the perceived drivers of unnecessary medical care

Although many physician participants praised CWC as a positive initiative that may help them address various concerns in the clinical encounter, when pressed, many also acknowledged that the campaign would likely fail to address the true drivers of unnecessary care. The analysis of the interview transcripts revealed that physicians identified three main perceived drivers of unnecessary medical care; these were time pressures in the clinical encounter, lack of knowledge about a patient’s potential pathway of care, and fear of litigation for not providing a service.

### Time pressure in the clinical encounter

Physician participants consistently reported that their work environment has immense time pressures, which limits their time with patients, and thus makes it difficult to engage patients in a conversation about their medical care. This seemingly contradicts earlier statements by physicians that they would like to engage patients in a dialogue about unnecessary medical care. Time constraints were a major contributor to providing unnecessary care:

“*If I’m really busy and I have ten people in the waiting room, and if I feel pressured and overwhelmed, I can say, ‘Yep, here is a requisition for the MRI, let’s get it done and move along*.” (P018)

Some physicians reported that there is less time to spend with patients in a hospital because of the number of patients waiting for care. As one physician noted:

“*Overcrowding for more* [medical services] *is a problem*.” (P008)

These statements indicate a major perceived driver of unnecessary medical care is the limited time available for the clinical encounter. Physicians report that there are too many patients to see in a day to spend adequate time conversing with them about their respective problems. Therefore, some physicians may provide a test or treatment to move the patient visit along. One physician provides a strong example of providing an unnecessary test to a patient to delay spending time on them at present:

“*It’s easier to order a test, and something that I think is not talked about but is done a lot is that we hide behind tests, and so we will state to the patient and their family ‘we are not exactly sure what’s going on but we are waiting on some test’ just to buy* [the physician] *some time. That test could be an x-ray of their left toe, and it has no impact. It’s just some time when you have no idea what’s going on, so it gives you something to hide behind*.” (P002)

Another physician commented further:

“*I think, in some of those areas* [time in the clinical encounter]*, it is going to be tough to address* [with CWC]*. That’s particularly in clinical care, where there is a lot of imaging, and it may not be as useful as people think...*” (P010)

Participants confirm that CWC should encourage physicians to engage their patients in a dialogue during the clinical encounter; however, many were unsure how they would implement it in their practice. Furthermore, if the physician is ‘not exactly sure what is going on,’ is under time constraints, and feels obligated to provide a service to a demanding patient, it is unclear whether the CWC will be beneficial.

### Uncertainty in the care pathway

Physicians may provide unnecessary care at the clinical encounter because they are uncertain what tests may be needed further down the care pathway. Therefore, physicians may order tests deemed unnecessary at the time to avoid stoppages further down their patient’s pathway of care. For example, many physicians do not want to see their patients held up because a specialist down the line of care requests a test that the physician thought was unnecessary at the time of referral. However, because the specialist has a history of ordering the test, the patient’s care is held up for the test results. Although pre-emptive ordering of unnecessary tests was perceived to be a major cause of providing unnecessary care, many physician participants stated that CWC does not address it:

“*One of the underlining principles is… one of my colleagues somewhere down the road is going to ask for* [tests]*. If this hasn’t been done, having a patient that hasn’t had a test in three years…* [surgeons] *cannot perform the surgery until it’s done. Surgeons are slow to cancel stuff that somebody might want sometime, and if it meant that the procedure would be cancelled or delayed and that was certainly the greater of the evil… The surest way to not worry about an abnormal test derailing your day is to do them*.” (P007)

Another participant explained:

“[Specialists] *may need additional information. They may need the x-ray services. They will tend to steer on the side of getting a test, even though it may be unnecessary, because they fear that they will not be able to get the patient referred*.” (P010)

These examples demonstrate the uncertainty a physician may have regarding their patient’s care pathway. Generally, many physician participants reported that they preferred a potentially unnecessary test done if there is a risk of delay further down the care pathway.

### Fear of litigation

Litigation over potential mistreatment was a prominent issue for physician participants. They stated that CWC does not address litigation problems. For example, one participant stated:

“*Concerns about ruling everything out and covering all their bases, concerns about litigation are missing* [from CWC].” (P003)

Due to some of the pressures to provide medical care, physicians often felt that they did not have enough information to say no to a potentially innocuous procedure:

“*I think litigation is a problem; you miss one neck… fracture or bleed in the brain you are going to court*.” (P008)

This lack of information becomes increasingly difficult when considered in the context of a demanding patient who may raise concerns that the physician may not have fully considered. Regardless of whether the concerns are well founded or not, there is some obligation to investigate. One participant explains:

“*Once the issue has been raised, it is difficult to back away unless you are 100% because you are responsible if you are wrong, and the test may have presented something*.” (P008)

Physician participants reported that CWC does not address their concerns over possible legal ramifications of not providing a test or treatment that may provide information about a diagnosis that would otherwise remain unknown. CWC does not provide any legal support for physicians if they follow its recommendations. Coupled with the pressure from demanding patients and uncertainty in the care pathway, fear of litigation puts immense pressure on physicians in the clinical encounter to provide medical care.

## Discussion

### Principal findings

The responses of physician participants provided several insights into perceived reasons for, and characteristics of, CWC to reduce unnecessary medical care. Participants, who included leaders of many medical specialty societies, reported that the time had come for the medical community to respond to existing pressures from patients, the public more generally, and government to address the issue of unnecessary medical care. In the absence of addressing these pressures, physicians considered themselves at risk of losing some of their autonomy, power and reputation. Their response was a campaign that focused on the clinical encounter to promote a discussion with patients about unnecessary care. Although participants supported the initiative as an alternative to government policies, they broadly agreed that CWC does not truly address many reasons why unnecessary care continues to be provided, including time pressures in addressing patient demands, uncertainty in a patient’s care pathway and fear of litigation. It is unclear how physicians will generate more time to engage in shared decision-making with the patient regarding why, in some instances, medical care should not be provided. Rather, the results here suggest that physicians preferred to opt for the safer route of providing care ‘just in case’ (for example, just in case an issue arises further down the patients’ care pathway).

### Strengths and weaknesses

This is the first study, to our knowledge, that qualitatively explores the perceptions of physicians about any CW campaign. The study included a wide range of medical professionals from primary, secondary and tertiary care. Therefore, perspectives from various types of healthcare providers were obtained. Furthermore, many participants were in leadership roles within their associations; this provided an excellent opportunity to receive informed insight into CWC. The insight serves as the foundation for our results and provides unique insight into the CWC campaign design rationale and features.

This study has several limitations that should be noted before considering its implications. First, the sample is limited in that the participants included were only those involved with the CWC, rather than physicians who were not involved in the initiative. Attempts were made to recruit members of medical specialty groups that did not have a ‘do-not-do’ list, but none agreed to participate. Had these groups participated, it may have provided a better-rounded perspective of CWC. The Quebec Medical Association also attempted to recruit a participant for the study, but was unable to recruit a member willing to participate in the interview in English. In addition, researchers and system leaders were not interviewed, which may have provided a more critical perspective to the analysis.

There was also limited experience with the actual application of CWC in the clinical encounter by the respondents. Although participants could provide valuable insights regarding the study’s objectives, there was only one participant that had an experience using the CWC material in a clinical encounter. Most physicians did not have experience implementing CWC in their practice. Instead, their perspectives focus on their experiences with providing unnecessary medical care and promoting CWC within their speciality. Their perspectives on CWC are perceptions based on their expert opinions of their clinical and working environment.

Additionally, we acknowledge that the results of qualitative research may be influenced by the bias of the researcher. In this study, the interviews and the analysis were conducted solely by the primary author, therefore heightening the risk of bias. Although we cannot eliminate preconceived perspectives, values or beliefs we did follow procedures to mitigate the risk of bias and safeguard the reliability and accuracy of results. For example, analytic memos were created to help guide analysis and maintain records. Furthermore, there were, at minimum, weekly in-person meetings and ongoing correspondence between the authors to discuss the findings throughout the analysis and write up. Although coding and themes were created by the primary author (ME), GR assisted in selecting quotes that were most representative of each category as well as provided a breadth of responses such that all participants were represented in the results.

### Findings in relation to other studies

Participants reported that patient’s demands, limited time in the clinical encounter, uncertainty in the care pathway and fear of litigation are prominent factors that influence a physician’s decisions to provide unnecessary medical care in the clinical encounter. A survey of Canadian and United States physicians found that many physicians believe unnecessary care is provided at least once a week due to these factors, with over half the physicians indicating that they would provide unnecessary medical care to a demanding patient [20]. Similarly, a large proportion of surveyed Canadian and United States physicians claim they did not have adequate time with patients or the clinical autonomy to meet patient needs [21]. Furthermore, other research highlights each of the drivers of unnecessary care identified in this study as contributors to unnecessary care. Investigation of patient preferences suggests that patient demand plays a small, empirically insignificant role in regional variation and it is physician preferences that dominate care decisions [3]. In a systematic review of barriers to and facilitators of implementing shared decision-making in the clinical encounter, which the CWC is advocating, time constraints were reported by physicians as the most important barrier [22]. Uncertainty in the care pathway is well established as a contributor to unnecessary medical care, particularly given the influence of supplier-induced demand [23], a well-known contributor to regional variation of healthcare [24, 25]. Finally, fear of litigation may result in defensive medicine, which may lead to provision of unnecessary care [26, 27].

### Implications for practice and policy

These drivers of unnecessary care are distinct and substantial and each requires targeted, evidence-based interventions to reduce the provision of unnecessary medical care. Unfortunately, as designed, the CW campaigns are ill equipped to address these issues. The finding that only one physician participant had talked to a patient about the CWC campaign and its messages in the care setting (and they had tried it only once), further supports the assertion that CWC is insufficient to reduce unnecessary care. Furthermore, the lack of uptake of the CWC approach in the clinical encounter suggests that it has not had any substantive impact on changing physician behaviour. This is consistent with other findings that physician behaviour is a significant contributor to unnecessary medical care [1]. Of further concern is that, in the present study, participant physicians did not identify themselves directly as contributors of unnecessary medical care. In other words, they see other physicians, rather than themselves, as contributing to the problem. If the average physician shares the view of the study participants, then they are unlikely to change their practice to reduce unnecessary care due to CWC. Previous research also found that United States physicians were reluctant to claim responsibility for high healthcare costs associated with unnecessary care; instead, many physicians identify pharmaceutical companies, insurers and lawyers as the most responsible groups for rising healthcare costs [28, 29]. If members of the medical community, including CWC designers and promoters, genuinely intend to reduce unnecessary care, then they will need to find ways to address these barriers to implementation.

Findings imply that policy-makers must be careful in creating policies that impact medical care delivery options because such policies may be perceived as not being in the patients’ best interest. Instead, participants believed governments will often create polices for financial reasons with the intent of reducing spending by reducing availability of care. Mistrust of top-down policies, may create political resistance from the medical community and a poor public perception of government intentions. Therefore, it may be in the best interest of policy-makers to work alongside the medical community as well as patient groups to promote micro-level policies that reduce unnecessary medical care at the point of care.

### Implications for research

The provision of medical care will often align with the preferences of providers and their patients, which regularly means more care [24, 25]. An important consideration for future research is how to sufficiently realign these preferences such that both parties are incentivised (in the broadest sense of that term) to decide upon appropriate care, which may often equate to less care. Considering results from this study along with previously described concerns, it is evident that CW campaigns, in general, do not realign these preferences in such a manner. If that remains the case, significant behaviour modification due to CWC is unlikely. Mainly due to the low quality of evidence, current research on shared decision-making does not allow firm conclusions on the most effective methods to improve adoption of shared decision-making; however, it does indicate that interventions including both patients and providers may be more effective than targeting either one alone [30]. Additional, high quality research on how shared decision-making may help reduce unnecessary medical care is a necessary first step to determining if the design of the CW campaigns will do more than address perceived pressures on the medical community. A descriptive analysis of CWC campaign material revealed that the patient material components do not meet minimal requirements as decision aids based on International Patient Decision Aid Standards [31]. This suggests that further development of patient materials is required to make CWC an effective shared decision-making tool. To improve CWC materials, it will be important to understand the patient’s perspective on the CW campaign’s design and features to assess whether it meets their concerns in the clinical encounter, including uncertainty in their care pathway. Understanding the patient perspective regarding what their role should be in shared decision-making, beyond the CW campaigns, is a much-needed next step for investigation.

## Conclusion

This study assessed key informant perspectives on CWC as an example of a CW initiative aiming to reduce unnecessary medical care. Using the perspectives of key informants involved in CWC, we sought to better understand reasons for the campaign’s design and which of its characteristics are expected to reduce unnecessary medical care. Our findings shed light on participants’ perspectives that CWC intends to address the pressures physicians felt to deliver unnecessary medical care, but they acknowledged that the campaign leaves many reasons for unnecessary medical care unaddressed. A concerning interpretation of the findings is that participating physicians did not attribute responsibility to themselves in the provision of unnecessary medical care. This finding is more concerning when considering that previous research regarding unwarranted variation indicates physician behaviour is a significant contributor to unnecessary medical care. If the aim of CW campaigns is to break the cycle of inappropriate care, then it will need to provide physicians and patients with the evidence and tools they need to know what the right care is and what is unnecessary. Physicians need to become better communicators and patients need to become more accepting of the evidence that indicates they are sometimes better off with less care. ‘Do-not-do’ lists are unlikely to address the pressures in the clinical encounter that lead to unnecessary medical care.

## Abbreviations

CW: Choosing Wisely
CWC: Choosing Wisely Canada
